# A case report of critical aortic stenosis diagnosed utilizing non-imaging continuous wave Doppler probe

**DOI:** 10.1093/ehjcr/ytae501

**Published:** 2024-09-14

**Authors:** Edward D Shin, Eugene Fan

**Affiliations:** Department of Medicine, Kaiser Permanente Oakland Medical Center, 3701 Broadway, 4th Floor, Oakland, CA 94611, USA; Department of Medicine, Kaiser Permanente Oakland Medical Center, 3701 Broadway, 4th Floor, Oakland, CA 94611, USA

**Keywords:** Aortic stenosis, PEDOF probe, Echocardiography, Case report

## Abstract

**Background:**

Aortic stenosis (AS) is the most commonly acquired valvular pathology in the western world. Aortic stenosis severity is typically evaluated with Doppler echocardiography. Evaluation of aortic gradients using standard Doppler echocardiography from the apical window may underestimate the true gradient due to misalignment of blood flow to the ultrasound beam and is often better evaluated from other imaging windows using a non-imaging continuous wave Doppler (NI-CWD) probe. Herein, we describe a unique case of AS being underestimated by dynamic acoustic shadowing from the apical window rather than beam misalignment.

**Case summary:**

The patient is a Hispanic 31-year-old male with a congenital bicuspid aortic valve who underwent a balloon aortic valvuloplasty at age 13. At age 31, the patient underwent a repeat transthoracic echocardiogram (TTE) that was largely unchanged from his prior TTE from 15 years prior. Notably on this TTE, there was acoustic shadowing of colour Doppler in the distal left ventricular outflow tract and aortic valve during systole. While gradients only suggested moderate AS, the degree of left ventricular hypertrophy was suspicious for more severe AS. Only by using the NI-CWD probe at the right sternal border were we able to identify very severe AS with a peak velocity of 6.5 m/s and a mean gradient of 100 mmHg.

**Discussion:**

In our specific case, dynamic acoustic shadowing of the aortic valve from the apical window obscured both imaging and Doppler signals. This acoustic shadowing was not present from the right sternal border with the NI-CWD probe, leading to an over 100% increase in aortic valve peak velocity and proper correction of AS severity. This allowed for expedited care and underscores the importance of such techniques.

**Learning points**

- Aortic stenosis severity is evaluated with Doppler echocardiography, but gradients can be underestimated from the apical window for various reasons.
- Non-imaging continuous wave Doppler from the right sternal border can be used to obtain more accurate reflection of severity of valve stenosis.

## Introduction

Aortic stenosis (AS) is the most common acquired valvular pathology in the western world, affecting as many as 2.5 million people over the age of 75 years.^1,2^ Timely diagnosis of severe AS is crucial for determining candidacy for aortic valve replacement. Aortic stenosis severity is primarily evaluated with Doppler echocardiography, including continuous wave (CW) Doppler ultrasound to measure aortic valve peak velocity, mean gradient, and aortic valve area (AVA) by continuity equation.^3^ Evaluation of aortic valve gradients using standard Doppler echocardiography from the apical window has been shown to often underestimate the true gradient slightly due to misalignment of blood flow to the ultrasound beam and is often better evaluated from other imaging windows including the right sternal border and suprasternal notch using a non-imaging continuous wave Doppler (NI-CWD) probe, also known as the PEDOF (pulsed echo Doppler flowmeter), Pedoff, linear, or standalone probe.^3^ Here, we describe a unique case report of critical AS with a peak velocity of 6.5 m/s seen only on NI-CWD from the right sternal border and was severely underestimated on standard apical Doppler echocardiography due to dynamic acoustic shadowing rather than beam misalignment.

## Summary figure

**Figure:**
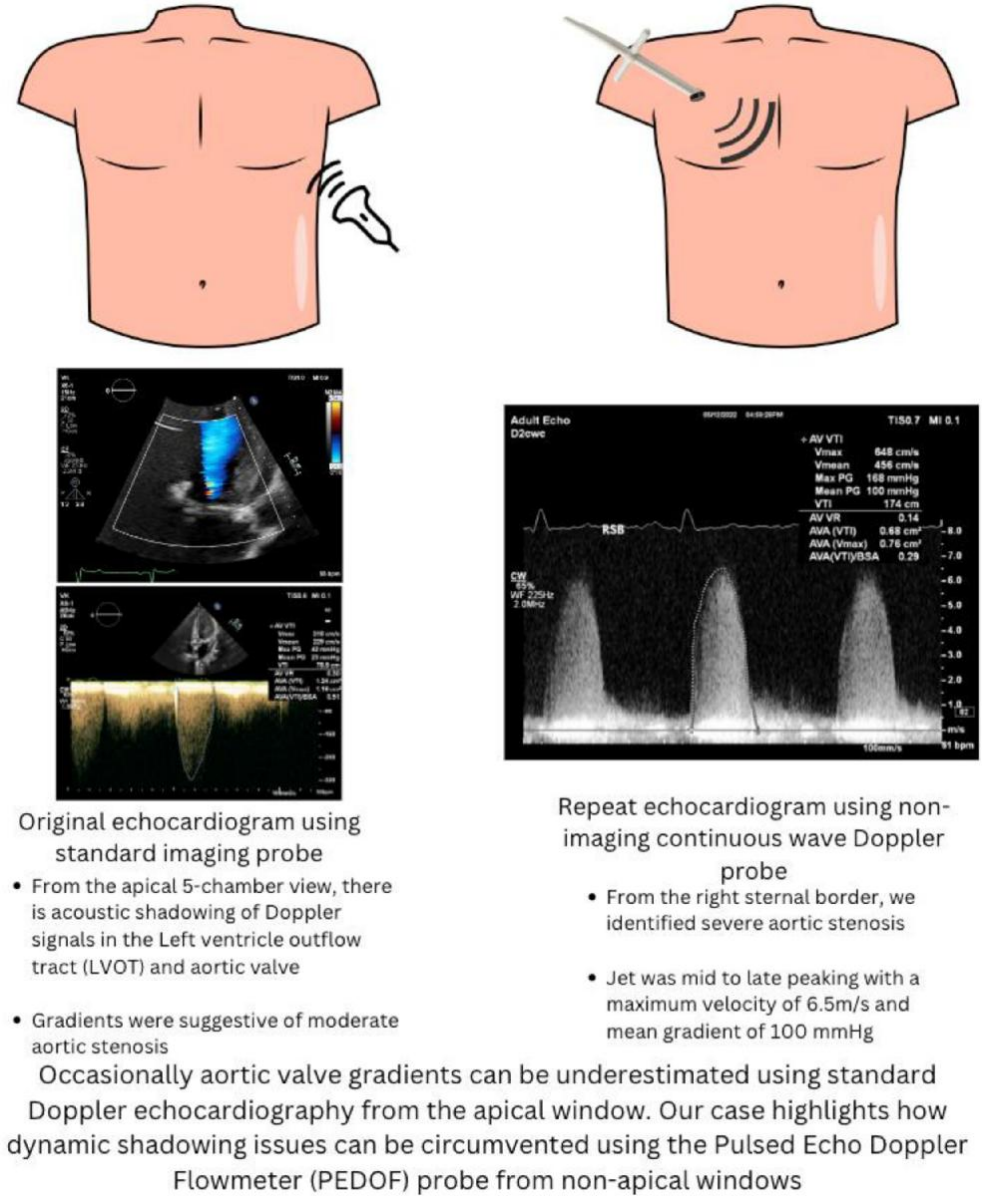


## Case presentation

**Figure 1:**
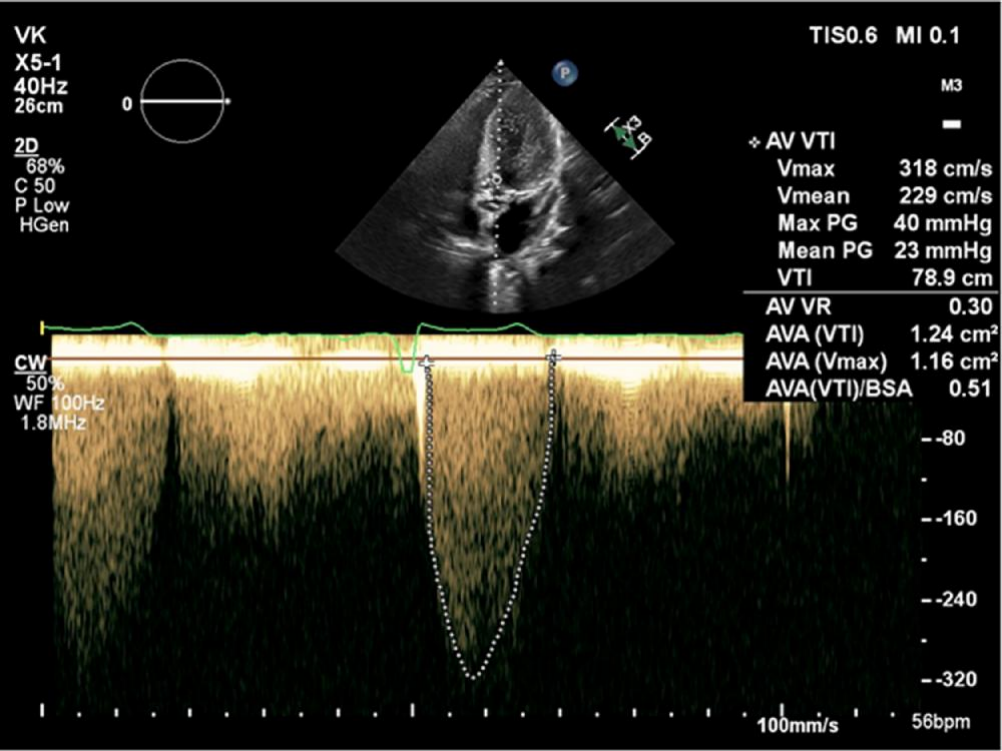
Transthoracic echocardiogram apical five-chamber view showing poor continuous wave waveform through the aortic valve.

**Figure 2:**
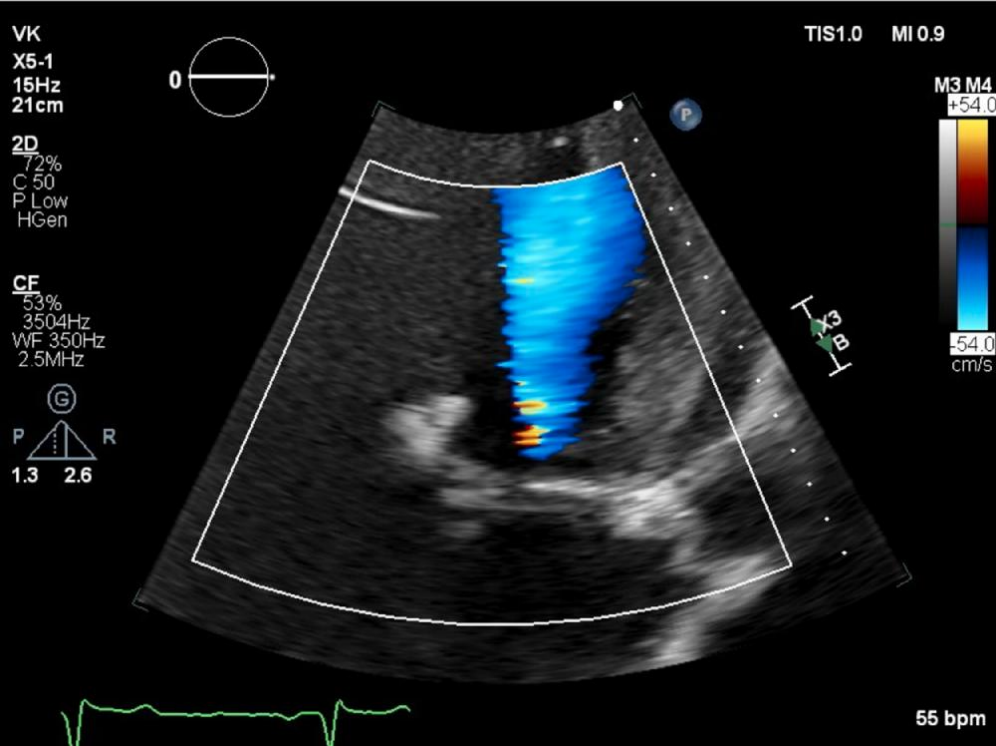
Transthoracic echocardiogram apical five-chamber view colour Doppler showing acoustic shadowing in the distal left ventricular outflow tract and aortic valve during systole.

**Figure 3:**
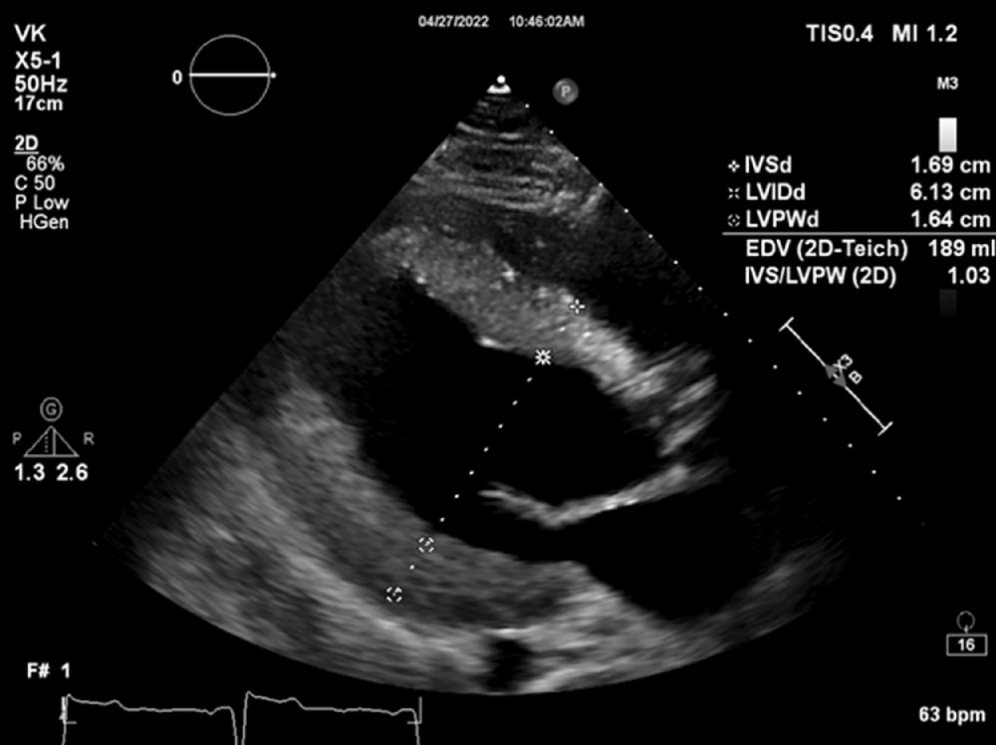
Transthoracic echocardiogram parasternal long-axis view demonstrates severe concentric left ventricular hypertrophy.

**Figure 4:**
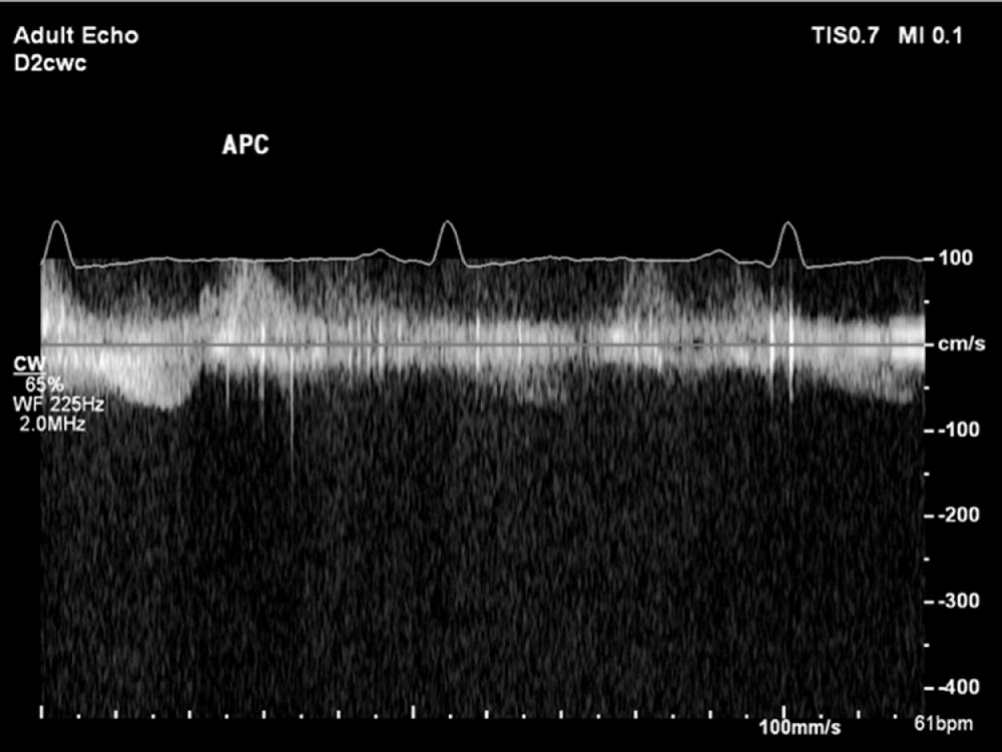
Repeat transthoracic echocardiogram using the non-imaging continuous wave Doppler probe from the cardiac apex shows poor continuous wave Doppler waveform.

**Figure 5:**
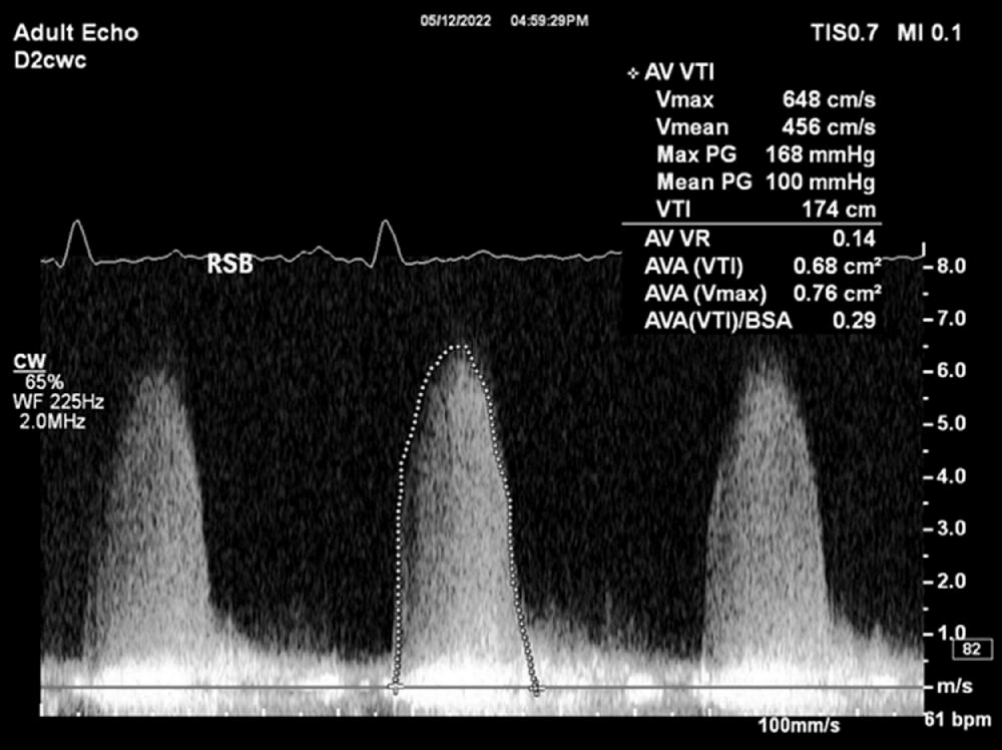
Repeat transthoracic echocardiogram using the non-imaging continuous wave Doppler probe from the right sternal border shows clear aortic valve continuous wave Doppler waveform.

The patient is a 31-year-old male with a past medical history of congenital bicuspid aortic valve found to have a heart murmur during infancy. The patient underwent a balloon aortic valvuloplasty for moderately severe AS at age 13. A follow-up transthoracic echocardiogram (TTE) at age 16 showed a thickened bicuspid aortic valve with a peak velocity of 3.1 m/s and a mean gradient of 21 mmHg from the apex, consistent with moderate aortic stenosis (images not available). He was not seen in a healthcare setting until presenting to an outpatient clinic at age 31 due to right groin pain. During this appointment, the patient was found to have a loud systolic murmur at the right upper sternal border on physical exam. The patient denied any cardiac symptoms. A follow-up TTE showed largely unchanged aortic valve gradients compared to 15 years prior (*V*_max_ 3.18 m/s, mean gradient 25 mmHg, and calculated AVA 1.24 cm^2^), but a poor spectral Doppler waveform (*Figure 1*), despite multiple attempts from the apical views. The aortic valve was not well visualized, and there was dynamic acoustic shadowing of colour Doppler in the distal left ventricular outflow tract and aortic valve during systole (*Figure 2*). While the gradients and calculated AVA only suggested moderate AS, the degree of concentric left ventricular hypertrophy was suspicious for more severe AS (*Figure 3*). A repeat TTE using NI-CWD probe was ordered. On the repeat TTE, aortic valve gradients were still not well seen from the apical window, both using the imaging probe (not pictured) and NI-CWD probe (*Figure 4*). However, the NI-CWD probe from the right sternal border, with the patient in a right lateral decubitus position, identified very severe AS with a maximum velocity of 6.5 m/s, a mean gradient of 100 mmHg, and a calculated AVA of 0.7 cm^2^ by continuity equation (*Figure 5*). The jet is mid-late peaking, also consistent with severe AS. The patient was urgently referred to cardiovascular surgery for aortic valve replacement. Several weeks later, the patient underwent aortic valve replacement with a 27 mm St. Jude mechanical aortic valve. Intraoperatively, the patient’s aortic valve was noted to be ‘heavily calcified and bizarre in appearance’. Unfortunately, no photos were taken of this valve. The patient has done well postoperatively, with follow-up TTE demonstrating a well-seated, well-functioning mechanical aortic valve (*V*_max_ 2.7 m/s, a mean gradient of 14 mmHg, and dimensionless index of 0.42).

## Discussion

Doppler echocardiography is the mainstay of assessing AS severity. Symptomatic patients with severe AS are at increased risk of adverse cardiac events and benefit from timely aortic valve replacement. Studies show an increased risk among patients with asymptomatic very severe (or ‘critical’) AS with a peak velocity of ≥5 m/s. An aortic velocity ≥5 m/s is associated with a >6-fold increased risk of cardiovascular mortality.^4^ As a result, current ACC/AHA guidelines provide a 2a recommendation for aortic valve replacement in this patient population.^5^ Underestimation of AS severity by Doppler echocardiography can occur due to misalignment of the ultrasound beam. This is especially true in older adults, where acute angulation of the aortic root can result in poor alignment from the cardiac apex. It is often recommended to use a NI-CWD transducer from various views as up to 21% of AS cases can be misclassified.^6^ The findings of Thaden *et al*.^7^ support this practice, showing the maximal velocity was most frequently obtained in the right parasternal window nearly 50% of the time, with up to 40% difference in calculated AVA.

While the PEDOF probe can often find slightly higher gradients based on aortic valve angulation, this is the first case described where AS severity was drastically underestimated without the PEDOF probe due to acoustic shadowing rather than angulation. It remains unclear whether the shadowing was from the left ventricle myocardium or an extra-cardiac structure, but it was present at multiple apical rib spaces. The shadowing was not present from the right sternal border, leading to an over 100% increase in aortic valve peak velocity and proper correction of AS severity from moderate to critical. This allowed for timely surgical intervention and avoided negative potential consequences of delayed diagnosis or invasive assessment. A trans-oesophageal echocardiogram (TEE) would have been the next step if the PEDOF study was still non-diagnostic. However, a TEE is an invasive procedure that carries some risk of sedation in the setting of critical AS, as well as the rare but potentially serious risk of damage to the throat and esophagus.

Our case highlights a unique presentation where a shadowing issue was circumvented using the PEDOF probe from multiple acoustic windows, allowing for expedited care. This highlights the utility of NI-CWD assessment in patients with a poor spectral Doppler waveform from the imaging probe, suspected discrepancy between valve appearance and gradients, or an angulated aortic valve, a common finding in elderly patients. The routine use of PEDOF imaging in suspected moderate or severe AS cases may help reduce underestimation of AS severity and ensure timely and appropriate intervention.

## Lead author biography


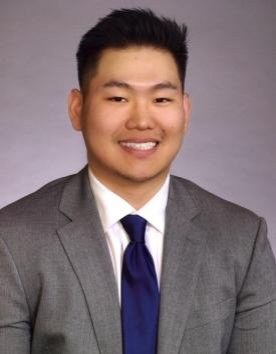


Dr Edward D. Shin graduated from the University of Wisconsin Madison Medical School with Research Honors. He is currently a third year internal medicine resident at the Kaiser Permanente Medical Center with plans for applying to a cardiology fellowship.

## Data Availability

The data underlying this article will be shared on reasonable request to the corresponding author.

## Notes

**Consent:** The authors confirm written consent for the submission and publication of this case report including images. The written consent form was compliant with COPE guidelines.

**Funding:** None declared.
